# Surgical Operations

**Published:** 1867-12

**Authors:** 


### ARTICLE XII.

### *Surgical Operations.*

From the weekly report of Mass. Gen. Hospital, published in *Boston Med. and Surg. Journal,* we take the following cases.

*Cancer of the Tongue.* By Dr. H. J. BIGLOW.—Patient, was an elderly man. Tumor of seven months growth situated underneath the right side of the tongue, about the size of an almond-meat, flattened, ulcerated, and somewhat raised from the surface. It was excised, and the hemorrhage, which was free from the whole cut surface, was arrested by the application of the solution of perchloride of iron.

*Staphyloraphy* By Dr. S. CABOT.—Patient was an adult male. Fissure extended through the hard and soft palates on the median line , anteriorly as far as opposite the first molar tooth. The soft parts being freely dissected up, the edges were pared and brought together by nine sutures.

*Salivary Fistula,* By. Dr. S. CABOT.—Patient, a boy. Fistula, following abscess, was situated about three-fourths of an inch behind the posterior edge of the parotid, discharging very little, except during meals, when there is a copious flow of saliva, to the great inconvenience of the patient. Incisions were made in the form of a letter H, the cross-bar of the H running through the fistula, the flaps were dissected up, one entirely cut away, and the edge of the other refreshed so as to get rid of the cicatrized edge of the fistula, and the edges united by sutures.

*Contraction of the Jaws.* By Dr. H. J. BIGLOW.—Patient a child, entered the hospital one year ago, with loss of the entire left cheek, by sloughing, after typhoid fever. The gums and teeth were exposed as far back as the coronoid process. The edges were pared and brought together after a long and tedious dissection, extending above the zygomatic arch, and downward upon the neck below the insertion of the tissues of the cheek into the lower horizontal margin of the jaw. The cheek, though tense, had united perfectly, by a horizontal cicatrix about three inches in length. Operation for contraction. The teeth were firmly locked, and the jaw immovable, as the result of the old and new cicatrices, so that a probe could be hardly introduced between the cicatrized tissue and the teeth. The dense tissues were gradually divided until the jaws were relaxed to admit a thin wooden wedge, by the aid of which, and other instruments, together with the successive divisons of the resisting bands as they presented themselves, the jaws at last yielded, and were separated to the extent of an inch. Dr. Bigelow remarked that with great care for months, about one-third of this advantage would probably be retained, which would be a great gain to the patient.

